# Mate assessment based on physical characteristics: a review and reflection

**DOI:** 10.1111/brv.13131

**Published:** 2024-08-22

**Authors:** Christopher D. Watkins

**Affiliations:** ^1^ Division of Psychology and Forensic Sciences, School of Applied Sciences Abertay University Kydd Building, Bell Street Dundee DD11HG UK

**Keywords:** face, voice, odour, body, movement, romantic relationships, sexual signalling

## Abstract

Mate choice, and sex differences in romantic behaviours, represented one of the first major applications of evolutionary biology to human behaviour. This paper reviews Darwinian approaches to heterosexual mate assessment based on physical characteristics, placing the literature in its historical context (1871–1979), before turning (predominantly) to psychological research on attractiveness judgements based on physical characteristics. Attractiveness is consistently inferred across multiple modalities, with biological theories explaining why we differentiate certain individuals, on average, from others. Simultaneously, it is a judgement that varies systematically in light of our own traits, environment, and experiences. Over 30 years of research has generated robust effects alongside reasons to be humble in our lack of understanding of the precise physiological mechanisms involved in mate assessment. This review concludes with three questions to focus attention in further research, and proposes that our romantic preferences still provide a critical window into the evolution of human sexuality.

## I. INTRODUCTION: MATE ASSESSMENT FROM DARWIN TO DIFFERENT DISCIPLINES (1871–1979)

**Fig. 1:**
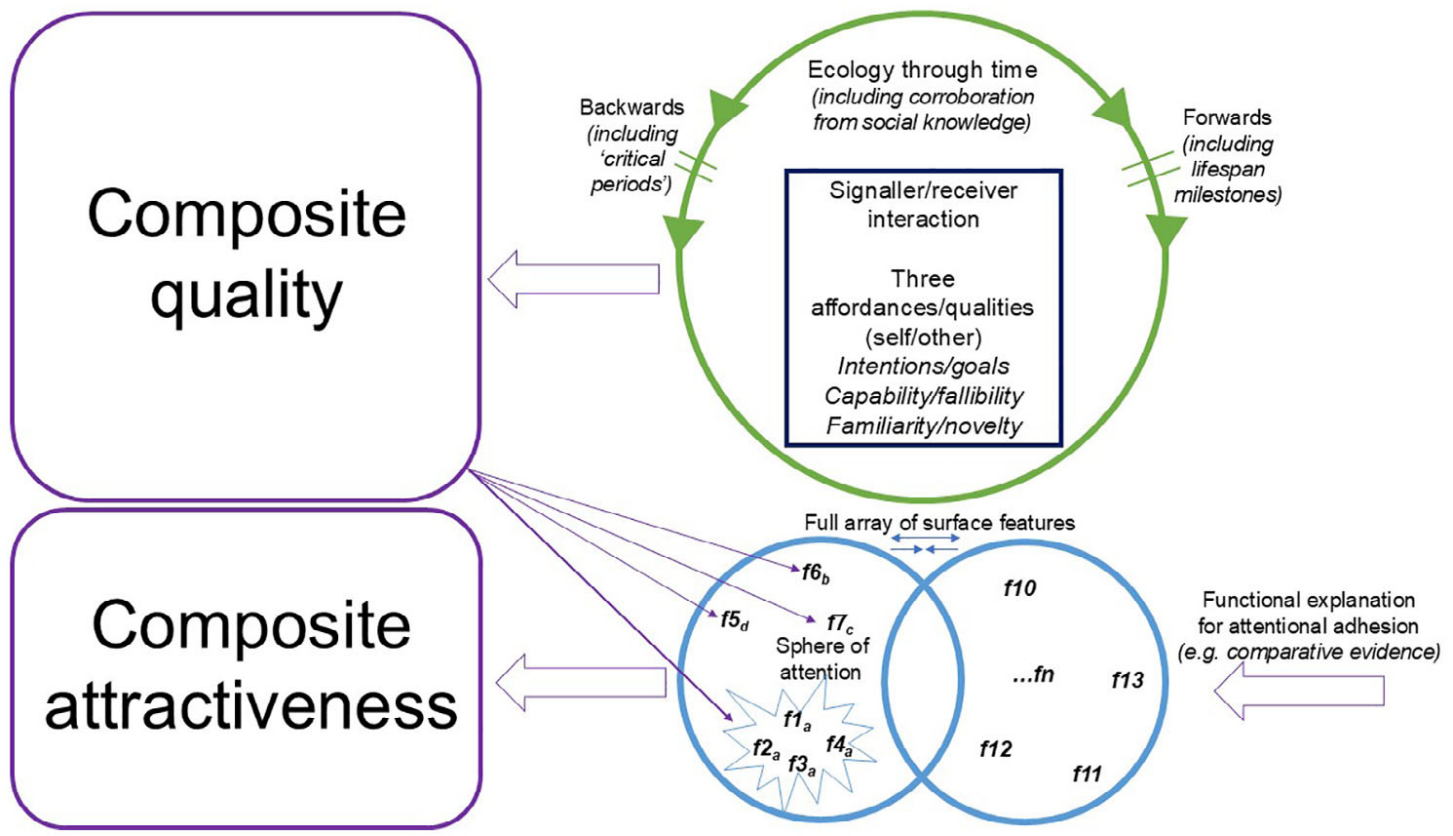
A model for mate assessment (univariate/component parts and multivariate/composite evaluations). Processes within the green circle enable the researcher to situate the individual judge (and their own qualities) within ecological (harshness/security) and lifespan perspectives (prior experience/key developmental milestones) as they make implicit judgements around three key qualities – (un)desirable intentions/goals, capabilities/fallibilities (e.g. related to dominance/health) and the extent to which they are familiar/novel within the environment (seen/unseen and/or an estimate of typicality/scarcity relative to the local population). Weightings attached to these three key qualities at each moment are then attached to an array of surface features (e.g. vocal, morphological, kinaesthetic) that the individual attends to (flexibly in light of the environment and social knowledge), where some features may cluster onto a single underlying quality (e.g. vocal and morphological indicators of size). This informs a holistic judgement of attractiveness.

When Charles Darwin (1809–1882) published *Sexual Selection and the Descent of Man in Relation to Sex* in 1871, he offered a remarkably prescient set of ideas and observations on mate assessment based on physical characteristics, drawing on other species, cultures, and offering speculations based on his own western social context in Victorian England. This framework for understanding courtship and reproduction in non‐human animal species is powerful to this day (e.g. Janicke *et al*., 2016). For example, in Chapters 19–21 and within his section on “sense of beauty” (Moore & Desmond, 2004), he argues for the importance of facial morphology (e.g. symmetry or regularity) and coloration, sex‐typical and exaggerated physical characteristics (e.g. hirsuteness), body size and shape, facial expression, voice, and musicality in mate choice. By explaining cultural standards *via* a universal framework, Darwin offered a unifying theory for cultural diversity in relation to sex (see square within Fig. 1), in contrast to some of his contemporaries who explained this diversity through a racist lens according to his biographers (Moore & Desmond, 2004). For example, while exploring the extent to which females exert more choice in courtship than males, and the importance of markers of creativity, intelligence, and status for males of “higher cultures” to attract female mates, he offers an early view on the extent to which the ecology shapes mate preferences. He quotes the German Philosopher Schopenhauer (Asher, 1871) on romantic love and courtship as far from arbitrary and within the remit and purview of the scientist:

The final aim of all love intrigues, be they comic or tragic, is really of more importance than all other ends in human life. What it all turns upon is nothing less than the composition of the next generation… It is not the weal or woe of any one individual, but that of the human race to come, which is here at stake.

Scholarly interest in aesthetics was of course not novel. Critical reflection on aesthetics is many centuries old as classicists debated the power of surface characteristics to afford given responses when they are encountered [see discussion of Sappho in Dion, Berscheid & Walster (1972) and of Aristotle in Todorov (2017)], ideas which would be developed by early physiognomists in the absence of scientific rigour (Todorov, 2017; conceptualised as rightmost square within Fig. 1 following empirical research). Darwin generally rejected the idea of a universal template such as a “golden ratio” for beauty, or a universal standard that was attractive to everyone in every context as outlined in an early review into evolutionary aesthetics (Grammer *et al*., 2003). However, dissemination of his theory of sexual selection coincided with the first scientific tests, to my knowledge, of social responses to physical characteristics and the positive attributions afforded by average or typical facial features (Galton, 1878; Jastrow, 1885; see square within Fig. 1). As Darwin's contemporaries and apprentices built on his theories, scholars of the early 20th century placed early emphasis on careful measurement in the study of individual differences in personality, intelligence, and morphology, their possible biological and cultural underpinnings, and their relation to social outcomes, while statisticians helped enhance the scientific status of psychology.

Darwin's theories of courtship and mate assessment would have a major influence on research on non‐human animals as the 20th century progressed. It is now well established that animal signalling occurs between potential mating partners and rivals for mates, based in part on coloration, ornamentation, “badges” of status, and traits exaggerated to a greater extent in one sex than another, influencing mating‐related competition and reproductive fitness [see, e.g. Andersson & Simmons (2006), Clutton‐Brock (2007), Emlen (2008), Santos, Scheck & Nakagawa (2011) and Weaver *et al*. (2018) for reviews]. This early research in nonhumans could be conceived of as converging with two main lines of post‐war research in humans that would go on to impact later theories: (*i*) early research into objective measures of the human sexual response; and (*ii*) early research from social psychology on the factors that underpin social stereotypes and categorical judgements of others, and the factors that help or hinder successful relationships within marriages, families, and social groups, such as “matching” or homophily [see, e.g. Reis *et al*. (2013) and Todorov *et al*. (2015) for discussion]. An explicitly evolutionary approach to human sexuality, drawing on research from other cultures and species, emerged in 1979 with the publication of Donald Symon's book *The Evolution of Human Sexuality* (Symons, 1979), followed by the first official conference of the Human Behaviour and Evolution Society in 1989, with a great deal of early research effort focussed on mate preference and mate choice in humans. This review reflects on the impact of this approach to our understanding of mate choice in humans, examining the morphological and dynamic physical characteristics involved in our assessments of potential mates (*what is typically attractive?*) followed by the role of the individual and their environment/circumstances in these judgements, and suggestions on how to move forward based on “knowing what we do not know” after 40 years of research effort. It highlights effects in the literature that are more *versus* less robust and the value of integrating different perspectives and lines of reasoning to inform an understanding of composite attractiveness. The next section will discuss research, predominantly from psychology, and the key theories and perspectives that inform our current understanding of mate assessment.

## II. EARLY THEORISING IN PSYCHOLOGY

Early progress in our understanding of mate assessment from a Darwinian perspective has been helped in no small part by the following key scholars and four major lines of enquiry. First, David Buss and David Schmitt, with pioneering cross‐cultural research demonstrated early evidence for universality in sex differences in mate preferences and sexual strategies (i.e. *between‐sex* variation; Buss, 1989; Buss & Schmitt, 1993), an effect replicated in a recent large‐scale cross‐cultural study, where women rank “good financial prospects” as more important in a romantic partner than do men, and men rank “good looks” as more important in a romantic partner than do women (Walter *et al*., 2020). Second, Steven Gangestad and Jeffry Simpson developed this line of reasoning to examine *within‐sex* variation in mate preferences and sexual strategies. Here, personal circumstances and the demands of a given ecology can lead to multiple, even contradictory, strategies and preferences that reflect the trade‐off over human evolutionary history between greater reproduction *versus* successful investment in those offspring over a delayed period of maturation (Gangestad & Simpson, 2000). Third, Martie Haselton and David Buss developed Error Management Theory to explain human perception in various contexts including romantic ones, where the least costly of an “optimistic” *versus* “cautious” strategy, from an evolutionary perspective, will shape behaviour in a given context, such as when making judgements or attributions of potential mates (Haselton & Buss, 2000; Haselton & Nettle, 2006; Johnson *et al*., 2013). Finally, a much deeper understanding of the origins of human romantic emotions can be obtained by drawing from research on other sexual behaviours studied *via* the same theoretical perspective, such as the pair‐bonding and sire choice/mate assessment hypotheses applied to romantic kissing and female orgasm (e.g. Watkins *et al*., 2019; Wlodarski & Dunbar, 2013; Wheatley & Puts, 2015). While the former hypothesis emphasises the role that romantic gestures and sexual behaviours play in maintaining long‐term close pair‐bonds, the latter, non‐mutually exclusive, hypothesis examines the role that these same behaviours play in assessing the putative biological quality of a romantic partner, particularly at early stages of a relationship such as courtship. The remainder of this article focuses on mate assessment as understood *via* research on social attributions of others based on their physical characteristics.

Research on social attributions based on physical characteristics has flourished by drawing on biological perspectives related to animal signalling, as physical characteristics send implicit signals to the observer about that individual's qualities as a potential mate, influencing that person's orientation towards them. In faces, first impressions of others are underpinned by two primary dimensions related to perceived valence/trustworthiness, and dominance (Jones *et al*., 2021; Oosterhof & Todorov, 2008), with a third attractiveness component observed in non‐standardised images typical of profile pictures (Vernon *et al*., 2014). An accumulation of theory‐driven research also demonstrates that social judgements based on appearance are made on a variety of trait dimensions (e.g. Blair, Judd & Chapleau, 2004; Eberhardt *et al*., 2006; Hammersmith & Biddle, 1994; Hassin & Trope, 2000; Mueller & Mazur, 1996; Todorov *et al*., 2005; Zebrowitz, 1997; Zebrowitz & McDonald, 1991), and from very brief interactions [see, e.g. Ambady & Rosenthal (1992) for a meta‐analytic review], with impressions of attractiveness and apparent personality formed rapidly and with little change after prolonged exposure (Willis & Todorov, 2006; see also Carré, McCormick & Mondloch, 2009; Todorov *et al*., 2005). Furthermore, attractiveness judgements are made implicitly, as demonstrated when such judgements are irrelevant to the experimental task at hand (Kramer *et al*., 2020; Liu & Chen, 2012; Luo, Rossion & Dzhelyova, 2019; Ritchie, Palermo & Rhodes, 2017). This has implications for how we orient ourselves towards, and choose, romantic and non‐romantic social partners (e.g. Andreoni & Petrie, 2008; Frieze, Olson & Russell, 1991; Karraker & Stern, 1990; Kenealy, Frude & Shaw, 1988; Little, Jones & Burriss, 2007; Marlowe, Schneider & Nelson, 1996; Wilson & Eckel, 2006; reviewed in Fiske, Cuddy & Glick, 2007; Judge & Cable, 2004; Langlois *et al*., 2000; Little, Jones & DeBruine, 2011*b*; Mazzella & Feingold, 1994; Olivola, Funk & Todorov, 2014; Rhodes, 2006; Todorov *et al*., 2015).

Beyond social attributions and the positive traits projected onto attractive individuals (Dion *et al*., 1972) such as being perceived as younger than their true age [Kwart, Foulsham & Kingstone, 2012; see Langlois *et al*. (2000) for a meta‐analytic review], physically attractive individuals are motivating to engage with at the neural (reviewed in Hahn & Perrett, 2014) and behavioural level (Kramer *et al*., 2020), and (facial) attractiveness is important in attention and memory, such as when measured *via* voluntary looking time (Leder, Mitrovic & Goller, 2016) and when remembering the location of, and engaging attention towards attractive women (Becker *et al*., 2005; Maner, Gailliot & Dewall, 2007), with attention a prerequisite to perception (Fig. 1). Moreover, biases in memory for attractive faces are underpinned by neural mechanisms involved in the encoding and processing of reward (Tsukiura & Cabeza, 2011), with attractiveness modulating face processing independent of both facial expression (Marzi & Viggiano, 2010), and facial distinctiveness (Wiese, Altmann & Schweinberger, 2014), and at very early stages of face processing (Chen *et al*., 2012).

Attractiveness influences behaviour at early stages of development as well as during early‐stage neural processing. For example, 2 to 15‐month‐old infants are sensitive both to symmetry and averageness and their preferences are shaped *via* visual experience with specific categories of face (reviewed in Damon *et al*., 2017). Other research suggests that symmetry preferences are present in 9‐year‐old children but not 5‐year‐old children, with smaller effects observed relative to adult preferences, which may point to a role of puberty and visual experience in face preferences (Vingilis‐Jaremko & Maurer, 2013; Fig. 1). Indeed, both stimulus and participant age may be important in the effects of attractiveness on behaviour. In memory research, effects of facial attractiveness on memory are strongest for young‐aged faces around the mid‐20s, with potential own‐age biases in face memory implicating a role for social goals in face processing (Lin *et al*., 2020), where younger‐age faces are differentiated on traits related to attractiveness to a greater extent than older‐age faces are (He *et al*., 2021). Biological perspectives on attractiveness propose that age, in and of itself, is important given its relationship to current and residual fertility, where facial ageing has a stronger negative effect on evaluations of other women (Ebner *et al*., 2018; Maestripieri *et al*., 2014). In sum, considering the suite of behaviours impacted *via* physical attractiveness, this review will discuss the extent to which physical cues are reliable in partner choice from a Darwinian perspective, focussing first on physical morphology as a guide to mate assessment. Within this review, the model in Fig. 1, can be used as a guide to this novel synthesis of over 30 years of research in this area, presented in a way that allows the reader to appreciate univariate, multivariate, and theory‐driven and data‐driven approaches to this topic, while potentially testing their own questions *via* these approaches in combination or isolation.

## III. MORPHOLOGICAL SIGNALS

Following proposals that the ability of all organisms to develop and maintain certain characteristics would denote good underlying “genetic quality” to observers (e.g. Thornhill & Gangestad, 1993), early research into human mate assessment focussed on the resemblance of physical features to a population average (averageness *versus* distinctiveness) and small anatomical deviations from perfect bilateral symmetry (symmetry *versus* asymmetry). Bilateral symmetry is correlated with a range of traits relevant to health, attractiveness, and personality, even though the size of these correlations is often small to moderate [e.g. Jones, DeBruine & Little, 2007*a*; see Rhodes (2006) and van Dongen & Gangestad (2011) for meta‐analytic reviews]. For example, while some studies have found a positive relationship between measured facial symmetry and self‐reported health (Shackelford & Larsen, 1999; Thornhill & Gangestad, 2006), and other putative health cues in women [diversity at non‐ major histocompatibility complex (MHC) loci; Lie, Rhodes & Simmons, 2008], others have found no relationship between facial symmetry and health assessed from medical records (Rhodes *et al*., 2001), even though the relationship between facial symmetry and perceived health (e.g. Fink, Grammer & Matts, 2006; Grammer & Thornhill, 1994; Jones *et al*., 2001; Penton‐Voak *et al*., 2001; Rhodes *et al*., 2001, 2007; Zebrowitz & Rhodes, 2004), and perceived attractiveness (Grammer & Thornhill, 1994; Jones *et al*., 2001, 2004; Penton‐Voak *et al*., 2001; Perrett *et al*., 1999; Rhodes *et al*., 2001) is robust.

Faces that are rated as average also tend to be rated as attractive (e.g. Light, Hollander & Kayra‐Stuart, 1981; Morris & Wickham, 2001; O'Toole *et al*., 1994). Research using computer graphic techniques demonstrated that average faces tend to be judged as more attractive than distinctive faces (e.g. Grammer & Thornhill, 1994; Langlois & Roggmann, 1990; Rhodes & Tremewan, 1996; Rhodes, Sumich & Byatt, 1999; Rhodes Simmons & Peters, 2005) and these findings are not due to the type of blending technique used to manufacture them (Little & Hancock, 2002). Moreover, familiarity (e.g. Batres, Kannan & Perrett, 2017; Peskin & Newell, 2004; Rhodes *et al*., 2001; Sofer *et al*., 2015) and typicality/representativeness of a population average (Lie *et al*., 2008) underpins attractiveness judgements of faces, at least in part. Studies that have increased the attractiveness of faces by increasing their “averageness” whilst keeping symmetry constant suggest that the attractiveness of average faces is not simply a consequence of the high correlation between averageness and symmetry, although symmetry does appear to contribute to the appeal of average faces (Jones *et al*., 2007*a*; Valentine, Darling & Donnelly, 2004).

Consistent with the proposal that averageness reflects a potential cue for underlying health, some studies have observed a positive correlation between averageness and measured health (Rhodes *et al*., 2001) and, in men, between facial averageness and other putative health measures, such as MHC heterozygosity (Lie *et al*., 2008). The positive effect of facial averageness on *perceived* health is also robust (e.g. Rhodes *et al*., 2001, 2007) and some studies have reported a positive correlation between averageness and other desirable personality characteristics linked to health, such as intelligence (Zebrowitz & Rhodes, 2004; Zebrowitz *et al*., 2002). Other research suggests that the appeal of average faces may be that average faces can be processed more easily by the visual recognition system (reviewed in DeBruine *et al*., 2007). Work on visual adaptation also suggests a degree of plasticity however, as our attractiveness perceptions of faces can be recalibrated by recent visual experience with new “groups” of faces [Rhodes *et al*., 2003*b*; see also Little *et al*. (2011*b*) for a review].

Evidence that averageness is related to attractiveness complements classic studies in social psychology which proposed that “familiarity breeds liking” [Zajonc, 1968; see Bornstein (1989) and Montoya *et al*. (2017) for meta‐analytic reviews], and research on non‐physical traits and mate choice, where similarity in interests, values, age, and education motivates repeated contact on online dating sites (reviewed in Finkel *et al*., 2012). While studies have suggested that facial averageness may be important for attractiveness (Grammer & Thornhill, 1994; Jastrow, 1885; Langlois & Roggmann, 1990; Light *et al*., 1981; Morris & Wickham, 2001; O'Toole *et al*., 1994; Rhodes *et al*., 1999, 2005), other work has demonstrated that highly attractive faces are non‐typical or extreme within a population and are *systematically* different from average faces (DeBruine *et al*., 2007; see also Perrett, May & Yoshikawa, 1994). For example, caricaturing highly attractive faces away from average makes them more, not less, attractive (DeBruine *et al*., 2007; see also Perrett *et al*., 1994). Exaggerated sex‐typical features are one type of extreme characteristic that many researchers have studied in relation to facial attractiveness.

Sexually dimorphic characteristics, or traits that are exaggerated to a greater extent in one sex than the other, are partly reflective of circulating oestrogen levels in women (Law Smith *et al*., 2006) and testosterone responses in men (Pound, Penton‐Voak & Surridge, 2009), although this latter relationship is complex and some null findings have been reported between men's baseline testosterone levels and their facial appearance (Neave *et al*., 2003; Penton‐Voak & Chen, 2004; Peters, Simmons & Rhodes, 2008; Roney *et al*., 2006). Data‐driven models of computer‐generated faces suggest that male facial attractiveness is driven by masculine reflectance/coloration and feminine face shape (Said & Todorov, 2011). This effect has been replicated in preference tasks based on real photographs (Carrito *et al*., 2016), with a partial replication for shape but not reflectance/coloration in data‐driven models of Asian faces, suggesting a degree of cross‐cultural similarity and variation across models (Nakamura & Watanabe, 2019).

In theory‐driven tests of experimental manipulations of shape in face photographs, there is good consensus that feminine *versus* masculine face shape is attractive in women (Little *et al*., 2001, 2002; Perrett *et al*., 1998; Penton‐Voak *et al*., 1999; Rhodes *et al*., 2003*a*). However, a combination of positive (Johnston *et al*., 2001; Jones *et al*., 2018; Penton‐Voak *et al*., 2001), negative (Burriss, Marcinkowska & Lyons, 2014; Carrito *et al*., 2016; Little *et al*., 2001, 2002; Perrett *et al*., 1998; Penton‐Voak *et al*., 1999; Rhodes *et al*., 2003*a*) and null effects (Cornwell *et al*., 2004; Scott *et al*., 2010; Stephen *et al*., 2012; Swaddle & Reierson, 2002) of masculinity *versus* femininity on men's facial attractiveness have been reported, with an overall null effect size in one meta‐analytic review (Rhodes, 2006). These mixed findings may partly reflect the combination of positive (health‐ and status‐related) and negative (dominant, anti‐social personality) traits inferred from masculine physical characteristics in men's faces and other modalities such as men's voices, bodies, and facial hair (e.g. DeBruine *et al*., 2006; Feinberg *et al*., 2006; Jones *et al*., 2010; Puts *et al*., 2007; Frederick & Haselton, 2007; Neave & Shields, 2008). Like attractiveness, there is a good degree of cross‐cultural consensus in perceptions of dominance from facial characteristics (Keating, Mazur & Segall, 1981; McArthur & Berry, 1987; Perrett *et al*., 1998; Rule *et al*., 2010) with such traits also inferred rapidly (Carré *et al*., 2009) and from very young ages (e.g. Keating & Bai, 1986; Pellegrini, 1995; Thomsen *et al*., 2011). Thus, sexual dimorphism may remain a fundamental dimension of study in the *perception* of potential romantic partners.

Evidence that attractiveness is influenced by similar parameters in different modalities strengthens the theoretical contribution of this literature. Cross‐modal signals may have been critical for the accurate assessment of mates among our ancestors, as demonstrated by research where increases in the attractiveness of women's face shape has a corresponding positive effect on those women's *vocal* attractiveness (Abend *et al*., 2015). Thus, common underlying qualities may shape mate choice. For example, somatotype (i.e. body shape and physique) explains a very high proportion of the variance in women's attractiveness ratings of men, with a positive effect of sexual dimorphism (muscularity) on men's attractiveness in western and cross‐cultural samples (reviewed in Dixson *et al*., 2014). Women make relatively quick judgements of men's somatotypes, with their distribution of attention to the upper and lower back of lean men suggesting assessment of a “V‐shape” figure across different relationship contexts (Dixson *et al*., 2014). Indeed, recent meta‐analyses indicate that men's muscularity has a robust positive effect on their reproductive potential and reproductive success across high‐ and low‐fertility populations, with other sexually dimorphic characteristics like facial masculinity only associated with measures of reproductive potential (Lidborg, Cross & Boothroyd, 2022). In women, medium breast size and breast firmness are attractive across different cultures, which researchers have interpreted as evidence for the attractiveness of residual fertility in women (Havlicek *et al*., 2017). As one body trait can indirectly affect the development of another body trait when the two are correlated (e.g. fat deposition and gain in body mass), multivariate approaches are also important in explaining the potential drivers of bodily attractiveness. These approaches suggest that preferences for single ratios like waist: hip ratio and body mass index may be a byproduct of heterosexual men's general preferences for slenderness, waist girth, and shapeliness (larger busts) in women (Brooks *et al*., 2015). Interestingly computer‐generated mate selection models, with small mutations visualised over generations as multiple body traits are manipulated independently, suggest that men's and women's ratings of female body attractiveness are almost perfectly correlated, and once a population‐wide average preference is established (i.e. selection above the 50th percentile), mate selection over generations may then work to optimise or exaggerate these traits away from average (Brooks *et al*., 2015). In sum, both univariate and multivariate approaches to bodily attractiveness complement and extend work on social judgements of facial morphology (Fig. 1).

Vocal parameters are also partly a reflection of somatotype, such as formants which explain variance in a person's height and mass (Pisanski *et al*., 2016), with these two traits also important in mate choice (e.g. Stulp *et al*., 2015; Tovee *et al*., 1998). Vocal characteristics have small but significant relationships with body shape when controlling for sex and age, such as perturbation (shimmer, jitter), which is negatively related to men's hip circumference and chest: hip ratio, and harmonics to noise ratios and formants, which predict women's waist: hip ratio (Pisanski *et al*., 2016). These parameters, in turn, are important in attractiveness judgements. For example, vocal attractiveness is negatively correlated with fluctuating asymmetry in both western and hunter–gatherer samples (Hill *et al*., 2017). Moreover, the similarity of an individual voice to an average voice, and regular/smoother voice textures (i.e. high harmonics to noise ratio) explain 40% of the variance in attractiveness ratings (Bestelmeyer *et al*., 2012). These parameters are strongly correlated with activity in voice‐sensitive auditory regions of the brain, where implicit judgements of vocal attractiveness also activate inferior frontal regions when listening to voice sounds without language (Bestelmeyer *et al*., 2012). These findings complement other research on the positive effects of averageness on the attractiveness of both faces and voices (Bruckert *et al*., 2010; Zaeske, Skuk & Schweinberger, 2020). On average, low *versus* high fundamental frequency is attractive in men's voices (Puts *et al*., 2016), with the opposite pattern observed for women's voices (Puts *et al*., 2011). The relationship between formant frequencies and vocal attractiveness is somewhat inconsistent across independent studies, however (reviewed in Hill & Puts, 2021). In sum, the effect of morphology on vocal characteristics is important in attractiveness judgements of potential mates.

While the evidence discussed thus far suggests that biological accounts are useful in explaining the attractiveness of specific morphological features of face, voice, and body, more direct manipulations of health arguably have greater utility in “good‐genes sexual selection” theories of attractiveness. For example, early studies into this issue suggested that the rated attractiveness of individual photographs predicted perceived health, but not measured health (Kalick *et al*., 1998). Other cross‐cultural research suggests that coloration, but not morphological masculinity, predicts ratings of men's facial attractiveness (Stephen *et al*., 2012, 2018). Indeed, when participants use computer graphic methods to increase the oxygenated blood colour of face photographs, this index of respiratory health increases perceptions of apparent health (Stephen *et al*., 2009*a*), as do manipulations of skin colour that denote high carotenoid levels, an index of fruit and vegetable consumption (Stephen *et al*., 2009*b*; Whitehead *et al*., 2012). Coloration indicative of high carotenoids is preferred across different ethnicities, both in faces and body parts but not control stimuli (scrambled versions of the same image; Ip, Lewis & Lefevre, 2019). Even small patches of (men's) facial skin rated high in apparent health are also rated as particularly attractive (Jones *et al*., 2004; see also Fink, Grammer & Thornhill, 2001; Fink *et al*., 2006), and patches of skin from MHC‐heterozygotes (an index of genetic quality) are rated as more attractive and healthier than patches of skin from MHC‐homozygotes, with these ratings positively correlated with attractiveness and health ratings from the whole face (Roberts *et al*., 2005).

Consistent with the proposal that morphology reflects a cue to underlying health, participants reliably alter faces on separate body mass index (BMI) and body fat dimensions to increase apparent health, suggesting that face shape is used as a subtle indicator of our underlying physiology (Stephen *et al*., 2017). However, both size and testosterone‐dependent characteristics have independent effects on appearance that generate cross‐sex and between‐sex differences, respectively [see Holzleitner *et al*. (2014) for discussion], where the latter may also have a combination of positive and negative effects on heterosexual women's assessments of potential mates, as discussed above. As such, there may be a more direct relationship between body size and judgements related to mate choice. Indeed, meta‐analyses demonstrate that people are very effective at estimating BMI from facial cues alone (*r* = 0.71; de Jager, Coetzee & Coetzee, 2018). Adiposity is a stronger predictor than sexual dimorphism of men's facial attractiveness, while both sexual dimorphism and facial adiposity predict women's facial attractiveness (Foo, Simmons & Rhodes, 2017). Although links between facial appearance and objective health measures were limited in this study (Foo *et al*., 2017), correlations between adiposity and objective health measures are generally well established (reviewed in de Jager *et al*., 2018). Thus, these traits influence mate assessment, either because attributions from facial adiposity convey potentially important information about people's health, or because people over‐infer health cues from minimal information (i.e. the face). It is worth noting here that health may be inferred not solely from the morphological or colour cues under study *per se*, but *via* the effects of poor *versus* good health on “leaking” certain emotions *via* subtle facial cues indicative of tiredness and/or negative emotional expression [see Henderson *et al*. (2016) for discussion]. Tiredness, for example, is an important factor in facial attractiveness perceptions as demonstrated in sleep‐deprivation experiments (e.g. Sundelin *et al*., 2017). As our current health status can of course change through time and with lifestyle (e.g. through lack of sleep and/or poor nutrition), the next section discusses the role that relatively dynamic signals play in mate assessment more generally.

## IV. DYNAMIC SIGNALS

Although there is some consistency in the evaluations we make of other people after longer exposure intervals (Willis & Todorov, 2006), there is of course a great deal of dynamism in our romantic interactions that can impact our assessment of potential partners. For example, eye gaze and emotional expression are critical in enabling us to decode others' attitudes and intentions (Haxby, Hoffmann & Gobbini, 2002). Both gaze direction and facial expression interact to modulate judgements of facial attractiveness (Jones *et al*., 2006) and emotional states (Adams & Kleck, 2005), while gaze direction and face shape interact to modulate judgements of others' dominance (Main *et al*., 2009). Cultural practices to enhance attractiveness (e.g. perceived femininity *via* makeup) are also a human universal (Kowal *et al*., 2022), impacting attractiveness perceptions, albeit to a smaller extent than their underlying facial morphology (Jones & Kramer, 2016). Vocal characteristics also change in line with the goals of the two interlocutors (reviewed in Leongomez *et al*., 2021), with even naïve listeners capable of inferring romantic interest by distinguishing between friends and romantic partners from very short voice clips taken from calls (Farley, Hughes & LaFayette, 2013). People also prefer the sound of speech directed to more *versus* less attractive individuals regardless of whether they are listening to their native language or unintelligible speech (Leongomez *et al*., 2014), with modulated pitch potentially important in courtship, particularly when we speak to attractive women (Leongomez *et al*., 2014). Other parameters may impact attractiveness *via* voice modulation. Here, while accentuating sex‐typical pitch may not have a direct benefit on vocal attractiveness, sex atypical pitch reduces vocal attractiveness (Fraccaro *et al*., 2013). Deliberate modulation of our voice may also impact courtship on other trait dimensions. For example, the fundamental trait dimensions implicated in social judgements of faces (Jones *et al*., 2021) may be perceived differently in individuals based on changes in pitch trajectories from very short vocal utterances (e.g. assertiveness, dominance, trustworthiness, and parallels to infant‐directed speech; Ponsot *et al*., 2018). Moreover, when people are asked to sound more attractive, confident, dominant, or intelligent, women's but not men's vocal attractiveness improves compared to their normal voice, while men, but not women, sound more confident to the opposite sex (Hughes, Mogilski & Harrison, 2014). Here, slowed speech and, in women only, lowered pitch/hoarse voice improves rated sexiness and attractiveness (Hughes *et al*., 2014). Collectively, a combination of how we express ourselves through our face and voice and how we direct our attention towards potential partners impacts mate assessment, potentially strengthening the positive effects of more static or morphological cues discussed earlier.

Chemical signals also add an element of dynamism to mate assessment. Although direct evidence for human pheromones may be deemed controversial, natural body odour may provide a marker both of genetic individuality and our current status, such as our diet, age, illness, emotional and physiological state, and potentially our personality traits (e.g. dominance and neuroticism), influencing women's attraction to male partners to a greater extent than men's, on average [see Ferdenzi *et al*. (2020) for a review]. Evidence that olfactory loss impairs the quality of a romantic relationship (Croy, Nordin & Hummel, 2014), points to the importance of social olfaction within romantic couples, particularly as relationships progress and these cues are integrated with others, as some have argued (reviewed in Groyecka *et al*., 2017). Indeed, real *versus* experimentally paired couples have more similar natural, but not fragranced, body odour, with natural body odour similarity positively related to men's relationship satisfaction and negatively related to women's relationship satisfaction (Allen *et al*., 2019). The pattern of these results is particularly noteworthy as within‐couple relationship satisfaction was highly correlated in this experiment, with odour similarity among couples driven by the “spicy/animalic” dimension (e.g. onion, spicy, animalic, heavy) but not the “sweet/milky” dimension of the olfactory lexicon (Allen *et al*., 2019).

Sexual selection perspectives have utility in social olfaction research, as odour is impacted *via* fertility‐ and/or mating‐related competition. For example, women's odour is more attractive during ovulation (Gildersleeve *et al*., 2012) and there is high agreement among men in their evaluations of women's body odour, which, in turn, is positively correlated with oestrogen and progesterone levels (Lobmaier *et al*., 2018). There is little evidence that putative health cues (MHC heterozygosity) underpin men's preference for fertile women's odour according to one study (Probst *et al*., 2017), and a recent meta‐analysis of the literature on MHC and odour preferences came to the striking conclusion that its relationship to mate preference is now far from clear across independent studies (Havlicek, Winternitz & Roberts, 2020). Context also impacts body odour in potentially adaptive ways. For example, induced sexual arousal from exposure to erotic films improves the attractiveness of women's body odour to men and, in turn, increases men's self‐reported sexual arousal, and the time they spend looking at, and the rated desirability of women, particularly if accompanied by sexual cues inferred *via* clothing and pose (Wisman & Shrira, 2020). In men, the general context of competition, rather than contest outcome, alters their body odour quality, by reducing the pleasantness, attractiveness, and intensity of their odour from pre‐match to post‐match, with some preliminary data suggesting that losing makes their body odour less attractive if the men were also in a negative mood state (Fialova *et al*., 2020).

The research discussed here suggests that characteristics accessible in odour add dynamism to mate assessment at different relationship stages. Recent reviews have suggested that extending measurements of social olfaction to samples other than the axilla, for example other areas implicated in close contact (hands and face), can improve our understanding of chemo‐signals and mate choice, as well as drawing from different disciplines and non‐western samples, and further exploration of the effects of contraception and fragrance use on odour (Ferdenzi *et al*., 2020). Early work on this suggests a similar level of intensity and attractiveness between different odour sources (axilla *versus* face/neck/scalp of head), with approximately 45% of the variability in odour evaluations accounted for by a valence dimension and a dimension related to organic decay (Ferdenzi *et al*., 2020). Here, unpleasantness rather than pleasantness may drive perceptions of odour attractiveness given the greater variability in descriptions used to evaluate the former *versus* latter (Ferdenzi *et al*., 2020). Different effects of artificial *versus* natural body odour on relationship dynamics are also noteworthy (Allen *et al*., 2016), if the cultural practice of fragrance use alters judgements of masculinity, gender, and personality, including prosocial traits (Ferdenzi *et al*., 2020). Further research on cross‐modal cognition, mate choice and chemo‐signalling may be fruitful, if for example, the effects of odour on self‐confidence influence attractiveness indirectly through movement [see, e.g. Spence (2021) for a review].

As implied by research on cross‐modal cognition, movement is perhaps the most direct contributor to our assessments of romantic partners in real time. Judgements of movement and posture reflect key dimensions of harmony and emotion and are important in how we gauge gender and the intentions of social partners such as the likelihood that they will reciprocate social effort (Cazzato, Siega & Urgesi, 2012). The brain appears to be specialised for the aesthetic appreciation of movements such as dance, with personal expertise in dance potentially generating stronger effects of dance on subsequent attractiveness judgements (Fink *et al*., 2021). Movement influences personality attributions in a consistent but *inaccurate* way, driven by its influence on our appraisals of attractiveness, masculinity–femininity, and emotional state, despite no corresponding relationship with self‐reports made on those same dimensions (Thoresen, Vuong & Atkinson, 2012). Trait ratings of movement are underpinned by two factors related to leisurely/focussed movement and a second factor of expansiveness, with this latter factor explaining a large proportion of the variance in trait judgements. Accordingly, increasing levels of both factors increases perceptions of extraversion, trustworthiness, and warmth (Thoresen *et al*., 2012). Although perceptions of traits derived from movement may not have a corresponding relationship with actual behaviours, some trait judgements made from dance may have a degree of truth and assist in mate choice and mating‐related competition, as inferences from individual and group dance may be made based on the good cognitive and physical skills required to execute and coordinate such movements effectively (reviewed in Fink *et al*., 2021). For example, men's neuroticism has a negative effect on women's perceptions of the attractiveness of men's dance moves (Weege *et al*., 2015). Moreover, judges accurately associate strength and dominance with “strong walkers”, classified *via* their grip strength, and women, but not men, perceive such men as more attractive than “weak walkers” (Fink *et al*., 2016). Attractive female dancers, in part, move based on hip swing, asymmetric thigh movement and intermediate arm movements (McCarty *et al*., 2017), while attractive male dancers do so *via* larger, faster, and more variable arm movements, particularly if they are physically strong (McCarty *et al*., 2013).

Even relatively subtle movements are important in romantic interactions during speed dating. For example, predictability of movement within a dyad, rather than similar (sway) movements, predicts long‐term but not short‐term interest during a speed date. This effect was observed independent of physical attractiveness which had a strong independent effect on willingness to meet and to pursue both a short‐term and longer‐term relationship (Chang *et al*., 2021). An (untested) illustration of predictable movement patterns within heterosexual courtship could be sex‐typical posture, gait, or expansiveness, if the information transferred between two time points is reliable even though the two individual's patterns of movement are uncorrelated. Finally, mate assessment can be influenced by cues within stimuli, as discussed earlier [see Henderson *et al*. (2016) for discussion]. For example, even though there is a very high correlation between attractiveness ratings of two‐dimensional (2D) and three‐dimensional (3D) images of female faces, women are perceived as more attractive in 3D (Tigue *et al*., 2012), perhaps because this representation provides further information and/or is inherently more rewarding due to its greater realism. In women, downward head tilt increases perceptions of “behavioural allure” (Sulikowski *et al*., 2015), while upward head tilt decreases perceptions of men's physical attractiveness and behavioural allure perhaps due to increasing perceptions of masculinity and traits related to dominance (Marshall, Bartolacci & Burke, 2020). Careful controls are important within stimulus sets [see, e.g. Sundelin *et al*. (2017) for effective protocols] in light of factors such as head tilt and a potential left‐side bias in facial attractiveness (Liu *et al*., 2021), where exposure of the left *versus* right cheek has a positive impact on the attractiveness of models and our evaluations of the products that they pose with, perhaps because the pose is associated with prototypicality (Park *et al*., 2021). Indeed, it may also be important to look at the attractiveness of individual features as different cultures appear to fixate on different regions within the face (Kawagoe, Kihara & Teramoto, 2020), albeit judgements of features/regions of the face (forehead, eyes, nose, mouth) are good predictors of whole face attractiveness (Liu *et al*., 2021). Research on within‐person variability in social impressions made from unstandardised images also points to the greater role of individual identity in social impressions of attractiveness, whereas individual *images* (of the same person) have a greater effect on social impressions of other traits important in mate choice (Todorov & Porter, 2014). At this point, mate assessment takes account of a given individual with their own goals, traits, and surrounding environment or culture.

## V. THE PERSONAL, SOCIAL, AND CULTURAL CONTEXT OF MATE ASSESSMENT

As acknowledged in an early review into evolutionary aesthetics (Grammer *et al*., 2003), Darwin was reluctant to ascribe universal standards of beauty to humans. Aesthetic preferences can differ systematically rather than randomly, however, in light of the person, their circumstances, and their surrounding environment or culture. Generally, scientific research on this topic has used strategic pluralism or “tradeoff theories” of mate preferences as a framework for understanding individual differences in mate choice (Gangestad & Simpson, 2000), in addition to biological markets theory, where “supply and demand” factors (see square in Fig. 1) influence preferences, mating strategies and the extent to which members of a given species can translate their preferences into choices [e.g. Noë & Hammerstein, 1994; see Maestripieri, Henry & Nickels (2017) for discussion and follow‐on commentary]. Attractiveness is generally a good proxy for reproductive fitness in humans, as it is correlated with mating success (Hughes, Dispenza & Gallup, 2004; Rhodes *et al*., 2005; Watkins *et al*., 2019), and, in one study, with the strength of the relationship between partnered women's preferences for facial cues and selection of those cues to putative biological quality in their current romantic partner (Wincenciak *et al*., 2015). One of the first studies on individual differences in mate preference demonstrated that attractive women had stronger preferences for a putative cue to underlying health (facial masculinity) relative to less‐attractive women (Little *et al*., 2001). This finding was supported by subsequent work using anthropometric measures of women's attractiveness (Penton‐Voak *et al*., 2003; Smith *et al*., 2009), and when testing the same question in other modalities (vocal masculinity; Vukovic *et al*., 2008, 2010), and with other facial metrics [symmetry; Jones *et al*., 2005; Little *et al*., 2001; but see Ekrami *et al*. (2021) for null effects in manipulations of 3D faces]. Recent replication among a large cross‐cultural sample suggests that the relationship between self‐rated attractiveness and masculinity preference is robust (Marcinkowska, Jones & Lee, 2021*a*), but critically may be limited to perceived attractiveness rather than physical condition *per se* given that another large‐scale replication suggested that the pattern of results does not generalise to third‐party attractiveness ratings of those same women (Docherty *et al*., 2020). In sum, there is good empirical support for the philosophical idea that beauty is in the eye of the beholder.

Early research into individual differences in mate preferences generated a great deal of subsequent research effort, primarily focussed on women's preferences for sexually dimorphic physical characteristics [see, e.g. Little *et al*. (2011*b*) for a review]. Examination of these traits was likely due, in part, to the corresponding alignment between perceptions of these characteristics (e.g. Perrett *et al*., 1998) and the primary dimensions on which we evaluate faces more generally [i.e. trustworthiness and dominance (Oosterhof & Todorov, 2008; Jones *et al*., 2021); see square within Fig. 1]. Thus, both data‐driven analyses, methods for manipulating physical characteristics on these dimensions, and theory on female mate choice and sexual strategies enabled researchers to test, and continue to test, a rich variety of predictions. Although a full review of environmental, contextual, and hormonal influences on mate preference is beyond the scope of this paper [see, e.g. Gangestad *et al*. (2019), Jones, Hahn & DeBruine (2019), Junger *et al*. (2018), Marcinkowska *et al*. (2019, 2021*b*) and Zietsch *et al*. (2015) for related discussion], such as demonstrated influences of environmental harshness/security and lifespan milestones on preference (Fig. 1), a few key findings are worth mentioning. First, the nature of the relationship an individual woman desires shapes her preferences for specific characteristics. This has been demonstrated such that women's preference for masculine men's faces are stronger when judging men's attractiveness for short‐term *versus* longer‐term relationships (Little *et al*., 2001, 2002; Penton‐Voak *et al*., 1999, 2003). This effect has been observed in other modalities such as voice (Puts, 2005), body shapes (Little *et al*., 2007), and *via* point‐light displays of biological motion (Provost, Troje & Quinsey, 2008), and is an effect replicated in a recent large‐scale longitudinal study of face preferences (Jones *et al*., 2018). Women's own self‐reported preference for short‐term sexual relationships is also related to their preference for so‐called “good genes” characteristics in men's faces and bodies (Ekrami *et al*., 2021; Provost *et al*., 2006, 2008; Quist *et al*., 2012; Smith *et al*., 2009; Waynforth, Delwadia & Camm, 2005). This same line of reasoning is related to the prediction that preferences for putative health cues [see, e.g. Foo *et al*. (2016) for a review of testosterone and immunity] would be stronger at times when women were more likely to conceive, and the benefits to offspring of “good genes men” would thus be greater, with research across different modalities supporting this assumption [see, e.g. Little *et al*. (2011*b*) for an early review and Marcinkowska, Galbarczyk & Jasienska (2018) for later research]. Recent non‐replications in longitudinal studies of large samples of women (Jones *et al*., 2018) challenge this assumption, where prior research may have represented false positive errors particularly if based on a between‐subjects' design [see Gangestad *et al*. (2016) for discussion]. Thus, while there is generally good evidence for effects of fertility and/or hormones on aspects of sexuality (e.g. Arslan *et al*., 2021) and social orientation, understood from the point of view of sexual selection theories [see, e.g. French & Meltzer (2020), Gangestad & Grebe (2017) and Jones *et al*. (2019) for discussion], the extent to which fertility shapes women's preferences for apparent “types” of romantic partner may be questioned by recent research, at least when testing for univariate relationships between fertility and a single physical characteristic.

Finally, as the literature on sexual selection theories of mate assessment developed, researchers started to turn their attention to the extent to which physical characteristics are integrated with social knowledge and recent experience. As such, researchers have tested the intuitive claim that experience and social knowledge shapes attraction by conducting empirical tests of the extent to which it leads individuals to “radiate beauty”, by overriding or moderating their physical appeal. For example, knowledge that individuals are attractive in the eyes of others leads to “copying” the preferences of third parties [e.g. Jones *et al*., 2007*b*; Little *et al*., 2015; Place *et al*., 2010; see Gouda‐Vossos *et al*. (2018) for a meta‐analytic review]. Moreover, we are inclined to copy the choices of people perceived as having good character (Chu, 2012), and the attractiveness of other people's physical characteristics is shaped by knowledge of their intelligence (Gao *et al*., 2017; Watkins, 2017), and romantic relationship history (Quist *et al*., 2012). Past experiences of cooperating or competing with individuals also influences the target's physical attractiveness (Faust, Chatterjee & Christopoulos, 2018; Kniffin & Wilson, 2004), with one of the few field experiments on this issue demonstrating that liking contributes a substantial proportion of the variance in physical attractiveness beyond initial attractiveness judgements made by mere strangers (Kniffin & Wilson, 2004). Indeed, we may even extrapolate our experiences onto unfamiliar individuals who share resemblance to previously encountered individuals. This proposal is supported by experiments where test faces are accompanied by positive *versus* negative emotional information, which has an effect in the predicted direction on judgements of novel faces that are morphed with those test faces (Verosky & Todorov, 2010). Collectively, first‐impression judgements based on physical characteristics are moderated in positive and negative ways by the experiences we have with those individuals, and the knowledge we garner about them from others.

## VI. FUTURE DIRECTIONS AND CRITICAL REFLECTION ON THE “STATE OF THE ART”

Darwin's theories of sexual selection and mate assessment were extremely prescient. When empirical psychology was but a nascent field, he managed to situate human courtship on the wider tree of life and put forward proposals on the characteristics important in mates that became well examined by scientists to this day. Modern research on mate assessment draws from diverse disciplines and examines a broader range of cultures than are typically studied within other fields of psychology. Technological developments have allowed us to visualise what Darwin could not at the time, by probing the contents of the unconscious mind and examining our internal representation of a face or voice, alongside other diverse methods, and paradigms beyond mere self‐report [see, e.g. Buss & Schmitt (2019) for a recent review], consistent with the importance of triangulation in science (Munafo & Davey Smith, 2018). Part of the purpose of this paper is to provide a “back to basics” reflection on the contribution of Darwin's seminal text to scientific thinking 150 years later. I suggest that there are three main questions to explore when evaluating the contribution of Darwin to our current understanding of human mate choice, with the current evidence synthesised within a model to act as a framework for future study (Fig. 1).

### (1) Are there limitations to the concept of “biological quality”?

Many early studies supported the proposal that attractiveness reflects a signal of that person's underlying biological quality, which would be advantageous in mate choice for the fitness benefits accrued to resulting offspring (e.g. Lie *et al*., 2008; Rhodes *et al*., 2001; Singh, 1993; Thornhill & Gangestad, 1993, 2006; van Dongen & Gangestad, 2011). However, as reviewed herein, the extent to which certain physical characteristics are objective measures of actual health is unclear (Foo *et al*., 2017), and the extent to which men's physical features afford indirect benefits for the offspring of female mates is also unclear across cultures [by improving offspring viability; see Lidborg *et al*. (2022) for a meta‐analytic review]. Thus, while certain physical characteristics may reliably be perceived as both healthier and more attractive, attractiveness may not be a reliable cue to quality. This conclusion is likely correct based on the typical age cohorts of both participant and target stimuli, and in research where time and resource constraints generally place an onus on establishing proof of concept based on a single physical metric and single proxy for health, normally measured at one time point. Universal access to modern medicine (in some societies) may obscure relationships between measured health and appearance or make them extremely difficult to observe in small samples. Indeed, research conducted to date on sexual selection for masculinity tends to examine reproductive potential as the outcome in low‐fertility populations and reproductive success as the outcome in high‐fertility populations, but not both (Lidborg *et al*., 2022). Longitudinal research may shed light on these issues, as could ethically designed experiments where the environment is somehow controlled such that natural exposure to stress or strain on the body is equivalent at baseline. This may have been the case for some retail workers exposed to a novel source of infection during the COVID‐19 pandemic, by virtue of their public‐facing role during this time. Indeed, the scientific claim that pathogen avoidance motivates a suite of approach‐avoidance behaviours is much less controversial, to the extent that we are prone to over‐infer disease cues as opposed to under‐infer their presence (Curtis, Aunger & Rabie, 2004; Tybur & Lieberman, 2016), consistent with error management theories of behaviour (Haselton & Buss, 2000; Johnson *et al*., 2013). These general psychological mechanisms may therefore influence mate assessment indirectly, such that a low‐health environment reduces our mating motivations more generally rather than our evaluations of a potential target (e.g. as originally demonstrated in Little, DeBruine & Jones, 2011*a*). Further research into the extent to which realistic or experimentally primed disease cues influence positive judgements of certain people more than others will help shed light on these issues, influenced by that person's perceived and/or objective health, and developing further explanations for recent null findings in this area [e,g. methodological limitations, see Tybur *et al*. (2020) for discussion].

When examining mating motives to improve reproductive fitness, and the extent to which these motives bear on our implicit and explicit assessments of potential mates, it is worth noting that some researchers have suggested that mate‐assessment mechanisms may have developed earlier in our evolutionary history than pair‐bonding mechanisms given that the former are observed among a wider range of early‐evolved species [see Wlodarski & Dunbar (2013) for discussion]. For certain contexts and research questions, pair‐bonding mechanisms may be psychologically more salient and inform partner choice accordingly. By contrast, corroboration of mate assessment theories may be contingent on sample characteristics, such as the attractiveness or demography of the cohort. Over development, all humans, independent of sexual orientation, can gain fitness benefits from strong and durable pair bonds (Opie *et al*., 2013), particularly in skills‐based economies where delayed maturation within our species is pertinent, and biparental investment to a period of independence is more valuable (Lawson & Mace, 2011). In sum, mate assessment still provides a theoretical framework to situate our species' mating system on the tree of life, and may still bear fruit through a combination of new methods and the study of individual differences in mate preferences, where we examine contexts where putative health cues are particularly salient, either directly in assessments of novel mates and current romantic partners, or in our evaluations of the situating context in which we encounter them, which could impact the nature of a given interaction.

### (2) Possible limitations if preferences do not translate to choices or can be explained by mechanisms beyond heterosexual mate choice

While there is some evidence that mate preferences based on physical characteristics are correlated with choosing those characteristics in actual romantic partners (e.g. DeBruine *et al*., 2006), this claim is still relatively under researched. While a relationship between preference and choice is important, *prima facie*, for sexual selection theories of human mate choice, we may risk throwing the baby out with the bathwater by diverting research attention away from understanding preference *via* a biological lens. Research on romantic preferences may be critical in understanding the “gatekeeper mechanisms” involved in sexual access, which will shape mating‐related behaviour within sections of the population in a different way compared to if such mechanisms are entirely absent and mate choice is indiscriminate. The “biological market” then provides a framework for understanding behaviour among potential mating prospects and competitors for those mates. Attractive individuals over time (Fig. 1) either do or do not “radiate beauty” through any combination of “matching” responsiveness to needs, and direct and indirect forms of mating competition. Indeed, partnership status is important in the study of attractiveness attributions, potentially over time (Karremans, Dotsch & Corneille, 2011; Watkins *et al*., 2017), where perceived relationship quality declines, on average, over time (Finkel *et al*., 2013). Preferences therefore can reflect authentic judgements of others at both the state and trait level.

Multivariate approaches and research on cross‐modal cognition may be important in establishing the relationship between preference and choice, as any small effects observed in this literature may still be theoretically important and have multiplicative effects on behaviour when combined across modalities, such as when examining sex‐typical traits in both morphology (e.g. Pisanski *et al*., 2016) and movement (Vacharkulksemsuk *et al*., 2016). Indeed, some multivariate analytical techniques have generated more substantive average differences between men and women in other areas (traits and temperament) than standard univariate approaches do (Del Giudice, Booth & Irwing, 2012). Speculatively, new research on singing may reveal a truly transformative trait in our judgements of a given individual. Research in this area could help us to make stronger inferences about the role of optimisation in mate choice *versus* trade‐offs between opposing traits. For example, vocal range is related to reproductive fitness in species of birds (reviewed in Catchpole, 1987) and may be optimal in women's mate choice if it advertises both prosocial and dominant personality traits simultaneously (e.g. *via* modulating pitch or formants; reviewed in Leongomez *et al*., 2021).

Second, a common valid criticism of evolutionary approaches to attractiveness judgements is the extent to which mate assessment theories have explanatory power if the appeal of specific physical traits generalises to contexts beyond heterosexual mate choice, such as when evaluating same‐sex social partners and/or when preferring similar traits in other species, or in nature or art (i.e. boundary effects). While some of the studies reported here demonstrate such context specificity [see, e.g. Little *et al*. (2011*b*) for a review] there are likewise many instances of effects generalising across sex and/or stimulus category. While testing for boundary effects with suitable control stimuli is of course important, general effects may not necessarily challenge theories of functional specialisation for human mate choice in the way that some suggest. For example, as discussed elsewhere (e.g. Damon *et al*., 2017) it is plausible that mate‐assessment mechanisms could generalise to other classes of stimuli or other social partners or species rather than *vice versa*. We are ultimately here to write and reflect on these issues today as our ancestors successfully navigated this foremost problem. As an illustration of this point, recent research on the attractiveness of musical chords is underpinned, in part, by its similarity to voice speech, in harmony and spacing (Bowling, Purves & Gill, 2018). Given the evolutionary trajectory of vocalisation to language and cultural tools for musical expression, it may be possible that we manipulate our environment or develop cultural tools as an extended phenotype for mate assessment, if certain stimuli have a shared deeper biological resonance.

### (3) The impact of technological advances and non‐evolutionary mechanisms on mate assessment

Evolutionary scientists often point to the “mismatch” between some of our evolved emotions and the environments we find ourselves in, in light of deep evolutionary time and the faster pace of cultural *versus* genetic change. Technology has shaped the process of human courtship even within the lifetimes of current 18–40‐year‐olds. Often implicit in the mate assessment literature is the idea that courtship is a “free process” involving an interaction between two interlocutors as opposed to one that might be conceived of as “regulated” either behind a screen, *via* a third party (friends or social networks), or *via* the appropriateness of translating certain preferences into declarations or actions in given environments. Such factors may alter how we pick and choose mates in and of themselves. Technology may amplify “evolved biases” as we use cultural tools to select mates from a wider pool. For example, following the original research on sex differences in mating strategies (Buss & Schmitt, 1993, 2019), real‐world data on contacts and responses suggest that these strategies are amplified on online platforms [i.e. more selective women and less‐selective men; see Finkel *et al*. (2012) and Sculley, Ritchie & Watkins (2021) for discussion], where women receive much more interest than men in general and some individuals may be very unlikely to receive replies let alone mutual romantic interest (Bruch & Newman, 2018). This may present an exciting opportunity to triangulate laboratory data with commercial and field data, to improve our understanding of and experiences with dating when such goals are pertinent.

Finally, the range of studies reviewed here demonstrates the utility of Darwinian theories in *a priori* tests of mate assessment in humans. As such, adaptive problems faced by pre‐modern societies and a range of species can explain the preferences and choices of humans today despite substantive changes in the sociology of human coupling. Socioeconomic factors, historical factors, and demography can of course account for patterns of human coupling in and of themselves, without necessarily requiring reference to evolutionary theory. For example, shifts to authoritarian systems limit the extent to which we can express our preferences, and wider socioeconomic factors may limit the extent to which preferences have a correlation with long‐term choices. Research from cultural evolution also has utility in accounting for the transmission of preferences *via* social contagion (see also Section V), and qualitative approaches can evoke a nuanced understanding of mate choice within diverse groups or choices driven by short‐term fashions or niche trends. Nonetheless, as illustrated in the proposed model (Fig. 1) there are opportunities for the field to move forward *via* continuation of an integrative and collaborative approach to this topic over 150 years since the publication of Darwin's text, integrating different research streams and testing alternate lines of reasoning within single research programmes. Perspectives from biological and cultural evolution, and wider social science, will shed light on whether cognitive systems for mate assessment are shaped through time or experience or are somewhat resistant to these factors. For example, within this model, one could use experimental or natural data to test whether in‐group biases based on physical attractiveness (e.g. typicality) are stronger or weaker in regions with homogenous/heterogeneous demography. The socioeconomic factors described above can also interact with biological factors, such as preferences for dominance and/or resource‐holding potential (see also Section III) and social contagion may be driven, in part, by the appearance of those we follow. For a good recent example of this integrative approach, Kowal *et al*.'s (2022) ambitious cross‐cultural study (*N* > 93,000) revealed both universality in attractiveness‐enhancing practices across >90 countries, as well as individual differences according to gender, age, ecology (historical infectious disease prevalence) and visual diet (social media usage). In sum, after much research effort, there is still a great deal of “low hanging fruit” and opportunities for new elegant study designs to test questions of great personal importance.

## VII. CONCLUSIONS

(1) Attractiveness is an important dimension in person perception and interaction. It is a trait inferred from different sensory modalities, and a judgement that varies systematically across different relationship contexts and in light of our surrounding environment. Mate assessment is therefore a highly sophisticated mental process, with an early phylogenetic and developmental basis and underpinned by some fundamental lower‐order cognitive processes, where the *perception* of various traits can shape later interactions and outcomes.
(2) There is good evidence for the importance of familiarity and typicality in attractiveness judgements across a range of paradigms. Future research should test whether an optimal degree of novelty is preferred considering recent experience (in experimental contexts) and examine interactions between mate‐assessment and pair‐bonding mechanisms at critical periods of development.
(3) We are far from a complete understanding of the physiological basis of mate assessment and partner choice however there is much low‐hanging fruit outlined here in both proof‐of‐concept research into interactions between physical traits and their effects on attractiveness judgements and more complex multivariate tests of the dynamic nature of romantic partner choice in various environments.
(4) By shedding some light on the explanations for various private feelings and emotions, responses to research on attractiveness can range from intrinsic curiosity to indifference from seemingly stating self‐evident truths, or anger at attempts to dehumanise aesthetic preferences by naturalising them. However, a standard social science model of mate assessment has an inherent problem in trying parsimoniously to explain attractiveness through general learning mechanisms when certain preferences are observed across species or developmental stages and even when observed consistently across *some* cultures with different norms and traditions. By viewing our romantic preferences as a construct that is infinitely mouldable *via* a given social elite, *this* perspective can dehumanise the experience of love and turn the question of what brought us onto the Earth as a parochial concern, actively preventing the growth of knowledge (Deutsch, 2011) on such a fundamental question.
